# Direct aperture optimization as a means of reducing the complexity of intensity modulated radiation therapy plans

**DOI:** 10.1186/1748-717X-4-8

**Published:** 2009-02-16

**Authors:** Maria Broderick, Michelle Leech, Mary Coffey

**Affiliations:** 1 Division of Radiation Therapy, School of Medicine, Trinity College Dublin, Dublin, Ireland, UK

## Abstract

Intensity Modulated Radiation Therapy (IMRT) is a means of delivering radiation therapy where the intensity of the beam is varied within the treatment field. This is done by dividing a large beam into many small beamlets. Dose constraints are assigned to both the target and sensitive structures and computerised inverse optimization is performed to find the individual weights of this large number of beamlets. The computer adjusts the intensities of these beamlets according to the required planning dose objectives. The optimized intensity patterns are then decomposed into a series of deliverable multi leaf collimator (MLC) shapes in the sequencing step.

One of the main problems of IMRT, which becomes even more apparent as the complexity of the IMRT plan increases, is the dramatic increase in the number of Monitor Units (MU) required to deliver a fractionated treatment. The difficulty with this increase in MU is its association with increased treatment times and a greater leakage of radiation from the MLCs increasing the total body dose and the risk of secondary cancers in patients. Therefore one attempts to find ways of reducing these MU without compromising plan quality.

The design of inverse planning systems where the beam is divided into small beamlets to produce the required intensity map automatically introduces complexity into IMRT treatment planning. Plan complexity is associated with many negative factors such as dosimetric uncertainty and delivery issues A large search space is required necessitating much computing power. However, the limitations of the delivery technology are not taken into consideration when designing the ideal intensity map therefore a further step termed the sequencing step is required to convert the ideal intensity map into a deliverable one.

Many approaches have been taken to reduce the complexity. These include setting intensity limits, putting penalties on the cost function and using smoothing filters Direct Aperture optimization (DAO) incorporates the limitations of the delivery technology at the initial design of the intensity map thereby eliminating the sequencing step. It also gives control over the number of segments and hence control over the complexity of the plan although the design of the segments is independent of the person preparing the plan.

## Review

### Introduction

Intensity Modulated Radiation Therapy (IMRT) is an advanced form of 3D radiotherapy. The non-intuitive nature of IMRT planning can sometimes lead to very complex plans. This review highlights the difficulties of overly complex plans and evaluates Direct Aperture Optimization (DAO) as a potential means of reducing this complexity.

Intensity Modulated Radiation therapy (IMRT) is a means of delivering radiation therapy where the intensity of the beam is varied within the treatment field. It is an advanced form of 3D conformal radiotherapy (3DCRT) which allows for more precise shaping of dose to the target and reduced dose to normal tissues. For example, in a study of 57 nasopharyngeal patients.[1] IMRT statistically decreased the dose to the parotid gland, temperomandibular joint, brain stem and spinal cord compared to conventional 3D radiotherapy. IMRT is of particular value for target volumes with concave or complex shapes with close proximity to radiosensitive normal structures[2] as it can allow dose escalation which would not have been possible with conventional 3D radiotherapy.

3D conformal radiotherapy is forward planned requiring the expertise of the planner to decide on the weights, beam orientation, use of wedges or compensators to achieve the desired dose distribution. IMRT, in contrast, is inverse planned in that the planner inputs the desired tumour dose and dose limits and the computer adjusts the beam intensities to achieve as close as possible to this desired outcome. With inverse planning, the dose distribution is broken up into different beamlets, with the computer tracing each beamlet through the patient producing the initial dose distribution. By altering the weights of individual beamlets the computer can accept these changes if it results in an improved distribution. This process is repeated for all beamlets during a single cycle. This is repeated until no further improvements are seen. At present this computerised process is very lengthy as the adjustments required to produce an acceptable plan are not intuitive as with forward planning[2].

IMRT can be delivered in many different ways; segmental, dynamic, arc or tomotherapy. Segmental IMRT (sIMRT) is where modulated field intensity is achieved by summing all the segments. The radiation is only turned on when the segment is in position, and is often known as step and shoot delivery. Dynamic MLC-IMRT is where the leaves are in continuous motion during each field. Although this method produces a more conformal dose distribution, a larger number of MU are required and there is more leaf transmission increasing the integral dose to normal tissue[3]. As the number of segments increases with sIMRT there is less of a difference between sIMRT and dynamic IMRT. Tomotherapy is a further means of delivering IMRT. It is a specifically designed unit similar to a CT which delivers IMRT with a fan beam.[2] Intensity modulated arc therapy is where the modulated intensity pattern is created as the gantry rotates around the patient.[4] The focus of this article is on sIMRT.

### Why reduce complexity?

The complexity of a plan is linked to the rate at which the fluence varies across each field. It is important however to avoid overly complex plans for several reasons.

First, IMRT requires more treatment fields than conventional radiotherapy and therefore a larger volume of normal tissue is exposed to low doses of radiation. Second, IMRT treatments are inefficient with respect to the number of MU which are necessary to deliver daily fractional dose leading to an increased leakage exposure. The more complex the intensity patterns, the higher the number of MU will be required to deliver the prescribed dose[5]. It has been demonstrated that one can reduce the number of MU greatly without affecting the plan quality[6] so the benefit of highly complex plans is questionable.

The increase in the number of monitor units relative to 3DCRT has led to concern about secondary malignancies with IMRT. Depending on the treatment energy IMRT treatments require 3.5–4.9 times as many MU to deliver a specified dose as compared to conventional treatments[7]. From this the conservative maximum risk of fatal secondary malignancy can be calculated to be 1.7% for conventional radiation, 2.1% for IMRT using 10 MV X-rays and 5.1% for IMRT using 18 MV Xrays[7].

However there is much uncertainty in these calculations of risk of secondary malignancies from the increase in MU[8]. In fact it is difficult in itself to calculate the increased dose in peripheral regions, as state of the art dose calculation algorithms in modern treatment planning systems are known to have uncertainties in the order of 10–20% of the local dose in low dose regions[9]. In attempting to determine the peripheral dose from segmental IMRT and compare it to an open field technique using TLDs it was found that the peripheral dose is increased by a factor varying from 1.2–1.8 compared to conventional radiotherapy, depending on energy and depth when delivering intensity modulated beams with sMLC [9].

The average number of MU per segment becomes very small for complex plans[10]. This may lead to dosimetric errors as the linearity of the output of the linear accelerator might not be sufficient to ensure accurate dose delivery. The size of some of the segments may be very small leading to further dosimetric uncertainty.

Treatment planning algorithms are less accurate in more complex IMRT treatments. Treatment fields which contain highly modulated intensity regions will be affected by low resolution pencil beam kernels. This was apparent in a study which compared two commercial planning systems. One of the systems not only underestimated the dose of the high intensity peaks by over 13% but it also overestimated the horizontal spread of the dose at the sides of the peak. This inadequacy of this treatment planning system was only relevant in highly modulated fields[11].

High dose rate, multiple beam segments, and low dose per segment have also been associated with the overshoot effect which generally occurs in the first and last beam segments due to the dose servo control system and causes an over and under dosage respectively[12]. It occurs in more complex plans where the errors relatively decrease by increasing MU per segment.

IMRT plans can result in hot spots distant from the tumour, from crossing high intensity beamlets. This can be counteracted by drawing 'help structures' at the area of the crossing but this may result in the hot spot appearing elsewhere. A positive side effect of controlling the intensity variation may be that these distal spots are less prevalent[6].

Decreasing the number of cycles in the optimization process has been shown to reduce dose dumping and MU[13]. This may be associated with decreased complexity.

The intensity distribution for each field is non-intuitive in highly complex plans and the complexity in general may increase the likelihood of human error. For example the verification of anatomical position through traditional imaging becomes difficult for multiple static segments of small size and varying intensity encompassing very little distinctive anatomy.[14] This will become less of a problem with more modern imaging techniques such as cone beam CT.

In addition to the dosimetric uncertainty and less intuitive nature of complex plans there is also increased machine and resource allocation required which may increase treatment times [15] and thus affect waiting lists.

A limitation in planning IMRT treatments is the time the optimization process takes. This can be reduced by decreasing the search space or number of variables. A common procedure would be to input set gantry angles for this reason. As computing power improves this will be less of an issue, but an important consideration is to use the computer power and time available appropriately. For example it may be more productive to use a more accurate dose calculation algorithm than using computer power calculating very complex plans, which may lead to inaccuracies.

### What is Direct Aperture Optimization?

IMRT can be forward planned or inverse planned. With forward planned IMRT the beams are first of all shaped to the target volume. Additional beam segments are then added and weightings are distributed between the larger beams and the segments in order to shape the isodose distribution. Forward planning IMRT has been shown to be superior to conventional forward planned tangential techniques in intact breast cancer although it is inferior to inverse planning techniques [16]

Aperture-based inverse planning IMRT adopts some of the features of forward planning and incorporates inverse planning into the process. Apertures are designed from the anatomical shape of the targets and conformal apertures are also designed that exclude critical structures. An inverse optimization is then performed to optimise the weights of the apertures within the provided dose constraints. This method therefore is a development of forward planning by using inverse planning software to optimise the weights of individual segments.

Direct Aperture optimisation (DAO), sometimes termed Direct Machine Parameter Optimisation, is another IMRT optimisation technique where similarly to aperture based inverse planning the apertures are identified during the planning process. However with this technique the apertures are not selected by considering the anatomical relationship between the target and critical structures. The planner inputs the dose constraints, beam angles, energies and number of apertures. With DAO the planner can also put a constraint on the minimum size of each aperture and place a lower bound on the weight [17]. The apertures are selected based on a few initial iterations and then the dose distribution is calculated for all fields. A large number of candidate apertures are sampled and either accepted or rejected depending on whether the plan is improved by adding the new aperture.

Beamlet based optimization divides a large beam into many small beamlets of about 1 cm^2^. Dose constraints are assigned to the targets and sensitive structures. Computerised inverse optimization must be performed to find the individual weights of this large number of beamlets. The computer adjusts the intensities of these beamlets according to the required planning dose objectives. Plans frequently fail to achieve the desired dose constraints and so clinical decisions have to be made as to which are most important and which can be relaxed. Once the optimal fluence map is decided upon there is a further leaf sequencing step. The optimized intensity patterns are decomposed into a series of deliverable MLC shapes made up of a number of basic beamlets with mathematically related weights/intensities. This typically results in a total number of leaf segments ranging from 60–100 in prostate only IMRT plans[15].

In converting the plan from the computer generated solution to deliverable segments, the dose distribution will degrade from that originally decided upon. In comparing beamlet based sIMRT for three commercial inverse planning similar performance was found in all three systems and IMRT plans tended to converge. The main differences in the IMRT plans concerned dose gradients outside the target, MU and segment number; demonstrating the impact of the sequencing step[18]. In another study it was found that the degradation in quality of the beamlet based optimized plan was solely attributable to the sequencing step as the plan quality prior to sequencing of the beamlet based plan was equal or less than the DAO plan. However after sequencing the beamlet based plan was worse than the DAO plan in all but 1 of the 11 cases.

DAO differs from beamlet based optimization in that it does not rely on the use of a segmentation routine (sequencing step) to select the initial leaf sequence as this step is incorporated into the original optimisation. Therefore with DAO the planner has direct control over the complexity of the IMRT plan. With DAO the treatment plan is optimized using a deliverable treatment solution. This avoids the plan degradation which can occur during the conversion of the ideal intensity map into a deliverable one at the end of optimization. The main purpose of direct aperture optimisation is to reduce the number of segments and MU required to treat a complex arrangement of targets and surrounding structures. The problems with large numbers of segments and high total MU have been discussed earlier.

### Clinical evaluation of Direct Aperture Optimization

Many of the dosimetric concerns associated with IMRT such as low MU per segment, high overall MU, and dosimetric uncertainties can be improved by controlling plan complexity. Direct aperture optimization is a method of controlling complexity that provides a significant reduction in the number of beam segments and MU required. However it also has the advantage over other methods of complexity reduction in that it eliminates the sequencing step which is associated with plan degradation. Studies which evaluated the use of DAO in the clinical setting are illustrated in table 1 along with details of how the plans were evaluated and compared.

**Table 1:** Clinical evaluation of DAO and the measures used in plan comparison.

**Author**	**Year**	**Participant numbers**	**Measures used in plan evaluation**
Van Asselen et al. [20]	2006	12 breast cancer patients	• V95 & V105 of TV • D99 & DD01 to measure homogeneity within TV • Dmax to heart • Mean dose of both lungs

Ahunbay EE et al. [19]	2007	15 breast cancer patients	• Uniformity index • Global uniformity index • V20 lung • V25 heart

Dobler et al. [22]	2007	10 hypopharyngeal patients	• Absolute values for DVH points which violated the Dose volume objectives(DVO). • D95 & D5 of PTV • V95 & VV107 of PTV • Daverage • Dose homogeneity measured by (D5–D95)/Daverage • D50 to parotid gland • Dmax spinal cord

Jones S et al. [21]	2008	11 head and neck patients	• Composite objective value i.e. the weighted sum of al the objective values • Conformity index

Ludlum E et al. [17]	2008	5 prostate and 5 nasopharynx patients	• V95-tumouir coverage • Multiple defined endpoints for sensitive structures • Conformity index • Homogenity index • Delivery time

When DAO was compared to 3D conformal radiotherapy, and beamlet based IMRT in 15 breast cancer patients it was found that DAO plans were equal to or better than those generated with 3D-CRT and standard beamlet IMRT[19]. DAO IMRT plans demonstrated statistically significant superiority over beamlet based optimisation in terms of breast dose uniformity (p = 0.003) and V20 lung dose (p = .008). There was also a trend for superiority of the DAO plans in terms of V25 heart dose although this did not reach statistical significance (p = 0.168). The MU for DAO were approximately 60% less than those for beamlet IMRT. Treatment time estimates were also calculated in this study and it was found that the DAO IMRT treatments would easily fit into the 15 min time slot but the beamlet based IMRT treatments would require slightly longer treatment slots of 17 min.

In another study which compared DAO and beamlet based IMRT plans of 12 breast cancer patients [20] the dose homogeneity within the target volume could be significantly improved with DAO compared to beamlet based IMRT although a large reduction in MU was not achieved with DAO in this study. This may have resulted from a low priority being placed on the constraint of the number of segments for the DAO plans and in fact in this case the beamlet based plans resulted on average with slightly less segments.

Jones at al.[21] compared DAO to beamlet based optimization in 11 head and neck cancer patients. DAO required 32% less time to calculate, 42% fewer MU and 35% fewer segments. It was interesting to note in this study that in 4/11 cases the conformity index was better for the beamlet based optimization. This was because the DAO plans produced a greater volume of reference (95% isodose) for these patients. Statistical significance was not calculated. This highlights the need for larger patient numbers in these studies as beamlet based optimization may still be necessary for more complex cases.

In a study of 10 hypopharyngeal patients, acting as their own control, no statistically significant difference was found for compliance to the dose volume constraints although the mean dose to the parotid was lower with the beamlet based plans compared to the DAO plans. Dose homogeneity within the PTV was superior for the DAO plans and they also required significantly less MU to deliver [22].

Ludlub et al.[17] compared DAO to beamlet based optimization in 5 prostate and 5 nasopharynx patients. They found that as the average number of segments decreased for both the DAO and beamlet based optimization the average conformal indices (COIN) values also decreased linearly but with a less steep slope for the DAO than the beamlet based plans. Therefore as the number of segments decreases there is less conformance as the slope is less steep with DAO, but for a given number of segments there is more conformance with DAO than with beamlet based optimization. DAO could create plans of similar quality yet with a significant reduction in the number of segments, requiring 3–5 times fewer segments reducing the delivery time for sMLC. With DAO, clinical requirements could be met for prostate patients with as few as 40 segments compared to 140–190 with beamlet based optimization. Clinical requirements could be met for nasopharyngeal patients with 50 segments using DAO plans compared to 140–200 segments with traditional beamlet based optimization.

The use of pencil beam algorithms when calculating IMRT treatments can lead to inaccuracies in difficult to calculate treatment geometries. To address this problem, Monte Carlo (MC) simulated dose data can be used with DAO in the optimization step. The optimized plan then undergoes a final dose calculation using MC. This is referred to as a MC DAO plan. This technique would be useful for planning small field IMRT cases for PTVs located within or adjacent to tissue inhomogenities. The optimized DVHs generated by the MC_DAO software are already a faithful representation of the final MC forward calculated doses as there is no leaf sequencing step required[22].

DAO can also be applied with collimator rotation, termed rotating aperture optimisation (RAO). Plans generated with RAO were found to be as good as or better than DAO, while maintaining a smaller number of apertures and MU than beamlet based optimization. RAO is less sensitive to tongue and groove effects than DAO. However delivery time is increased due to the collimator rotation speed[23].

### How much can you reduce segment number before compromising on plan quality?

Reducing the complexity of IMRT has been the focus of this review, but it has to be noted that there is a limit to the degree which the complexity of the plan can be reduced before severely affecting plan quality. This was demonstrated in prostate patients where it was found that for DAO plans with 20 segments the conformal indices values dropped drastically when compared to plans with 40 or greater segments confirming the indication for a threshold for the minimum number of segments[17]. It was again demonstrated by Craft et al.[6] where they investigated the trade off between treatment plan quality and the required number of MU.

The smallest width of any aperture is the width of an MLC leaf and the minimum length of any aperture is determined by the minimum step size along the direction of travel of the MLC leaves. The quality of an IMRT plan can be improved by increasing the number of degrees of freedom. Therefore increasing the number of apertures per beam should improve plan quality. Reducing the step size from 10 mm to 1 mm demonstrated continuous improvements in the objective function value and led to steeper dose gradients between the target and critical structures[24]. However this is at the expense of increasing plan complexity and treatment times. When Jiang et al. investigated the effect of increasing the number of apertures on objective function, isodose distribution and DVH for seven patients they found that although the number of apertures dramatically affected the objective function initially this curve reached a plateau after a certain number of apertures. They found few dosimetric gains in increasing the number of apertures per beam angle beyond nine [25]. Therefore although increasing the number of apertures may lead to slight improvements in objective function values this may not translate into significant clinical improvement beyond a certain number of apertures. Where this plateau occurs would differ depending on the complexity of the relationship between the target and critical structures along with the shape of the target. For simple cases with a convex tumour volume and a separation between the tumour and OAR there may be little improvement beyond three apertures per beam angle, while for larger more complex tumours particularly where the target is more concave in shape, the objective function converge more slowly as the number of apertures is increased and often nine of more apertures are required per beam angle[26].

### Other methods of reducing complication of IMRT plan

Other methods have been suggested to increase delivery efficiency and reduce MU without significantly affecting plan quality. In a study of 9 patients (including head and neck prostate and brain) it was found that by using intensity limits during inverse planning it was possible to reduce the total MU without compromising the clinical acceptability of the plan. MU reductions up to 38% were observed[27].

Matuszak et al.[28] looked at putting penalties on the cost function to reduce beam complexity and increase delivery efficiency. They compared three types of plans, those with a baseline cost function, plans with a baseline cost function employing maximum beamlet intensity limits and plans with beam modulation penalties added to the cost function, which penalised intensity map variation or variation from a filtered version of the optimized beams. All techniques yielded improvements over the baseline cost function. These plans were tested clinically on 3 head and neck patients, 3 prostate patients and 3 brain patients. In all methods an acceptable plan was reached while reducing MU substantially. Although each method has merit as a tool to reduce beam complexity, the penalty placed on plan intensity map variation consistently produced the most delivery efficient plans with fewest computations.

Smoothing parameters as either part of the optimization process or post optimization have been used to reduce the complexity in IMRT. Although those applied post optimization usually result in degradation of the plan quality, it has been shown that a smoother fluence can result in a reduction in dose to the healthy tissue and again that a reduction in fluence complexity is strictly correlated with a reduction in MU. Increasing the smoothing parameter has been shown to have an impact on the accuracy of delivery[29,30].

### Summary

It is important to reduce the complexity of IMRT plans as much as possible as overly complex plans deliver unnecessarily high MU and excessive radiation leakage. There is also more dosimetric uncertainty associated with highly complex plans as well as increased pressure on resources. Therefore a balance needs to be found between plan complexity and optimal dosimetry. Forward planning provides better dosimetry than 3D but is inferior in general to inverse planning approaches. Aperture based planning takes on some of the elements of forward and inverse planning. DAO differs from aperture based planning in that it is an entirely inverse planned approach which incorporates the restrictions of the treatment machine into the optimisation process. Many approaches have been taken to reducing the complexity of IMRT plans such as setting intensity limits, putting penalties on the cost function and using smoothing filters. DAO incorporates the treatment machine restrictions into the optimisation process thereby eliminating the sequencing step which has been associated with plan degradation. DAO has been associated with a reduction in MU in a number of clinical situations but further research needs to be conducted to establish the optimum number of segments for targets and OARs of varying geometry as it may not be possible to reduce segment number and MUs in the more difficult geometries.

## Conclusion

Due to increased pressure on resources, dosimetric uncertainties and leakage of radiation, treatment plans with very small fields, low MU per segment and high overall MU are not optimal. It is necessary therefore to look at ways of reducing the complexity of beamlet based IMRT treatment plans. It is also desirable to reduce the discrepancy between the optimal treatment solution and the deliverable treatment solution following segmentation. Many approaches have been taken to addressing these issues. This review discusses the potential role of DAO in attempting to reduce the number of field segments while achieving a similar dose distribution.

## Abbreviations

IMRT: Intensity Modulated Radiation Therapy; MLC: Multi leaf collimator; MU: Monitor Units; DAO: Direct Aperture optimization; 3DCRT: 3D conformal radiation therapy; sIMRT: segmental IMRT; sMLC: static MLC; TLD: thermo luminescent dosimeters; MC: Monte Carlo; RAO: Rotating Aperture optinization; OAR: Organ at risk

## Competing interests

The authors declare that they have no competing interests.

## Authors' contributions

MB conceived and drafted the manuscript. ML critically reviewed/revised the article. MC drafted the abstract. All authors read and approved the final manuscript.

## References

[B1] Poon I, Xia P, Weinberg V, Sultanem K, Akazawa C, Akazawa P, Verhey L, Quivey JM, Lee N (2007). A treatment planning analysis of inverse-planned and forward-planned intensity-modulated radiation therapy in nasopharyngeal carcinoma. Int J Radiat Oncol Biol Phys.

[B2] Taylor A, Powell ME (2004). Intensity-modulated radiotherapy – what is it?. Cancer Imaging.

[B3] Nicolini G, Fogliata A, Cozzi L (2005). IMRT with the sliding window: comparison of the static and dynamic methods. Dosimetric and spectral analysis. Radiother Oncol.

[B4] Galvin JM (2006). Alternative methods for intensity-modulated radiation therapy inverse planning and dose delivery. Semin Radiat Oncol.

[B5] Mohan R, Arnfield M, Tong S, Wu Q, Siebers J (2000). The impact of fluctuations in intensity patterns on the number of monitor units and the quality and accuracy of intensity modulated radiotherapy. Med Phys.

[B6] Craft D, Suss P, Bortfeld T (2007). The tradeoff between treatment plan quality and required number of monitor units in intensity-modulated radiotherapy. Int J Radiat Oncol Biol Phys.

[B7] Kry SF, Salehpour M, Followill DS, Stovall M, Kuban DA, White RA, Rosen II (2005). The calculated risk of fatal secondary malignancies from intensity-modulated radiation therapy. Int J Radiat Oncol Biol Phys.

[B8] Kry SF, Followill D, White RA, Stovall M, Kuban DA, Salehpour M (2007). Uncertainty of calculated risk estimates for secondary malignancies after radiotherapy. Int J Radiat Oncol Biol Phys.

[B9] Wiezorek T, Voigt A, Metzger N, Georg D, Schwedas M, Salz H, Wendt TG (2008). Experimental determination of peripheral doses for different IMRT techniques delivered by a Siemens linear accelerator. Strahlenther Onkol.

[B10] Verhey LJ (2002). Issues in optimization for planning of intensity-modulated radiation therapy. Semin Radiat Oncol.

[B11] Petric MP, Clark BG, Robar JL (2005). A comparison of two commercial treatment-planning systems to IMRT. J Appl Clin Med Phys.

[B12] Grigorov GN, Chow JCL, Barneett RB (2006). Dosimetric limitations and a dose correction methodology for step and shoot IMRT. Physics in Medicine and Biology.

[B13] Reddy NM, Mazur AK, Sampath S, Osian A, Sood BM, Ravi A, Nori D (2008). The potential for dose dumping in normal tissues with IMRT for pelvic and H&N cancers. Med Dosim.

[B14] Baker CR, Hardy V (2003). Provision of IMRT in the UK. Part 1: A review of planning, delivery and related technologies. Journal of Radiotherapy in Practice.

[B15] Ludlum E, Xia L (2008). Comparison of IMRT planning with two-step and one-step optimization: a way to simplify IMRT. Phys Med Biol.

[B16] Rongsrivam K, Rojpornpradit P, Lertbutsayanukul C, Sanghangthum T, Oonsiri S (2008). Dosimetric study of inverse-planned intensity modulated and conventional tangenital techniques in breast conserving radiotherapy. J Med Assoc Thai.

[B17] Shepard DM, Earl MA, Li XA, Naqvi S, Yu C (2002). Direct aperture optimization: a turnkey solution for step-and-shoot IMRT. Med Phys.

[B18] Georg D, Kroupa B, Georg P, Winkler P, Bogner J, Dieckmann K, Potter K (2006). Inverse planning – a comparative intersystem and interpatient constraint study. Strahlenther Onkol.

[B19] Ahunbay EE, Chen GP, Thatcher S, Jursinic PA, White J, Albano K, Li XI (2007). Direct aperture optimization-based intensity-modulated radiotherapy for whole breast irradiation. Int J Radiat Oncol Biol Phys.

[B20] Van Asselen B, Schwartz M, Van Vliet-Vroegindeweij C, Lebesque JV, Mijnheer BJ, Damen MF (2006). Intensity-modulated radiotherapy of breast cancer using direct aperture optimization. Radiother Oncol.

[B21] Jones S, Williams M (2008). Clinical evaluation of direct aperture optimization when applied to head-and-neck IMRT. Med Dosim.

[B22] Dobler B, Pohl F, Bogner L, Koelb O (2007). Comparison of direct machine parameter optimization versus fluence optimization with sequential sequencing in IMRT of hypopharyngeal cancer. Radiat Oncol.

[B23] Bergman AM, Bush K, Milette MP, Popescu IA, Otto K, Duzenli C (2006). Direct aperture optimization for IMRT using Monte Carlo generated beamlets. Med Phys.

[B24] Milette MP, Otto K (2007). Maximizing the potential of direct aperture optimization through collimator rotation. Med Phys.

[B25] Zhang G, Ziping J, Shepard D, Earl M, Yu C (2005). Effect of beamlet step-size on IMRT plan quality. Med Phys.

[B26] Jiang Z, Earl MA, Zhang GW, Yu CX, Shepard DM (2005). An examination of the number of required apertures for step-and-shoot IMRT. Phys Med Biol.

[B27] Jiang Z, Shepard D, Earl M, Naqvi S, Yu C (2004). The asymptotic limit of the number of field segments for IMRT using direct aperture optimisation. Medical Physics.

[B28] Coselmon MM, Moran JM, Radawski JD, Fraass BA (2005). Improving IMRT delivery efficiency using intensity limits during inverse planning. Med Phys.

[B29] Matuszak MM, Larsen EW, Fraass BA (2007). Reduction of IMRT beam complexity through the use of beam modulation penalties in the objective function. Med Phys.

[B30] Giorgia N, Antonella F, Eugenio V, Alessandro C, Filippo A, Luca C (2007). What is an acceptably smoothed fluence? Dosimetric and delivery considerations for dynamic sliding window IMRT. Radiat Oncol.

